# Genetic Mapping of Head Size Related Traits in Common Carp (*Cyprinus carpio*)

**DOI:** 10.3389/fgene.2018.00448

**Published:** 2018-10-09

**Authors:** Lin Chen, Wenzhu Peng, Shengnan Kong, Fei Pu, Baohua Chen, Zhixiong Zhou, Jianxin Feng, Xuejun Li, Peng Xu

**Affiliations:** ^1^State Key Laboratory of Marine Environmental Science, Xiamen University, Xiamen, China; ^2^College of Fisheries, Henan Normal University, Xinxiang, China; ^3^Henan Academy of Fishery Sciences, Zhengzhou, China; ^4^State Key Laboratory of Large Yellow Croaker Breeding, Ningde Fufa Fisheries Company Limited, Ningde, China; ^5^Laboratory for Marine Biology and Biotechnology, Qingdao National Laboratory for Marine Science and Technology, Qingdao, China

**Keywords:** common carp, QTL, GWAS, head size, head length, linkage mapping

## Abstract

Head size is important economic trait for many aquaculture fish which is directly linked to their carcass yield. The genetic basis of head size trait remains unclear in many widely cultured fish species. Common carp (*Cyprinus carpio*) is one of the most widely studied fish due to its importance on both economic and environmental aspects. In this study, we performed genome-wide association study using 433 Yellow River carp individuals from multiple families to identify loci and genes potentially associated with head size related traits including head length (HL), head length/body length ratio (HBR), eye diameter (ED), and eye cross (EC). QTL mapping was utilized to filter the effects of population stratification and improve power for the candidates identification in the largest surveyed family with a published genetic linkage map. Twelve SNPs showed significant for head size traits in GWAS and 18 QTLs were identified in QTL mapping. Our study combining both GWAS and QTL mapping could compensate the deficiency from each other and advance our understanding of head size traits in common carp. To acquire a better understanding of the correlation between head size and body growth, we also performed comparisons between QTLs of head size traits and growth-related traits. Candidate genes underlying head size traits were identified surrounding the significant SNPs, including *parvalbumin*, *srpk2*, *fsrp5*, *igf1*, *igf3*, *grb10*, *igf1r*, *notch2*, *sfrp2.* Many of these genes have been identified with potential functions on bone formation and growth. *Igf1* was a putative gene associated with both head size and body growth in Yellow River carp. The teleost-specific *igf3* was a candidate head size related gene, related to both HL and HBR. Our study also indicated the importance of Igf signaling pathway for both growth and head size determination in common carp, which could be potentially used in future selective breeding in common carp as well as other species.

## Introduction

Head size related traits such as head length (HL), head length/body length ratio (HBR), eye diameter (ED) and eye cross (EC) are not only important morphological indicators for fish taxonomy, but also play important roles in fish behaviors and environmental adaptation like locomotion. Head size is also important economic trait in fish because of the direct effect on carcass yield. Dog is another representative species with various strains distinguished by head size. HL was regarded as growth related trait (Fu B. et al., 2016). ED and EC are responsible for vision, and may have impact on feeding, predator avoidance and so on. ED and EC has also been shown a positive correlation with body weight (BW) and body length, hence can be used for indirect selection for other commercial traits (Jin et al., 2012). Because of the great value of head size in biology and economics, researchers have attempted to unravel the genetic basis of head size determination for many years, with the final goal of the development of molecular markers suitable for phenotype prediction and eventually used in molecular marker-assisted breeding (MAS). However, understanding the associations between effect loci or genes and head size related traits is still at the early stage. As commercial traits, it is of great value to investigate the genetic architecture responsible for head size traits with the final goal to produce fish with extreme big or small head for ornamental or economic reasons.

Genome-wide association study (GWAS) and quantitative trait loci (QTL) mapping have been proved powerful in the identification and characterization of genes influencing complex traits (Fu Z. et al., 2016; Zhu et al., 2016). A huge effort has been invested using QTL and GWAS strategies with growing number of molecular genetic markers in aquatic animals. Most of them were on the topics of growth (Gutierrez et al., 2015; Peng et al., 2016), immunity (Vallejo et al., 2014; Xin et al., 2015), environmental adaptability (Jin et al., 2016; Wang et al., 2017), and also some focused on the genetic basis of body shape (Carmelo et al., 2016; Jing et al., 2016). In fish, several reports have already identified genomic loci or regions putatively involved in head size. QTLs associated with HL, ED, and EC were reported in a common carp gynogenetic line (Liu et al., 2009). HL, HBR, ED and EC related QTLs were also identified in German mirror carp (Wang et al., 2010; Jin et al., 2012). Multiple regions associated with HL, head width and head depth were reported in catfish (Geng et al., 2016). Many kind of molecular markers were used in previous studies, including amplified fragment length polymorphism (AFLP), simple sequence repeats (SSRs). With the development of sequencing technology, single-nucleotide polymorphisms (SNPs) are increasingly used in genetic studies. Most of the previous studies only reflect the genetic content of a limited number of markers, result in a failure to cover the whole genome, especially for the species with complex genomes, for instance, common carp, leading to difficulties in achieving accurate mapping of the related traits. In addition, lack of genomic and genetic maps makes it even more difficult for some species to conduct genetic analysis and localize gene precisely. In consideration of all these problems, it’s particularly important to improve marker density and the genome resources.

Common carp (*Cyprinus carpio*) is one of the most important aquaculture species with a long domestication history and is widely distributed from Asia to Europe. There are abundant populations and domesticated strains in China, including Yellow River carp, Hebao red carp, Oujiang color carp, Songpu mirror carp, as well as many hybrid strains. The genetic diversity and allotetraploidized genome of common carp lead an increasing interest in the studies of this special species (Xu P. et al., 2014). Great efforts have been made on genetic and genomic studies of common carp over the past decade. Abundant omics data have become available, including the comparative exomes study of *Cyprinus carpio* and *Danio rerio* (Henkel et al., 2012), the complete genome assembling and annotation (Xu P. et al., 2014) and the full-body transcriptome and proteome resource (Kolder et al., 2016). Besides, a large number of genetic markers have been developed, especially the SNP markers, which were used for the production of common carp 250 K SNP array with markers evenly distributed across the reference genome with an average spacing of 6.6 kb (Xu J. et al., 2014). Furthermore, a large number of genetic maps have been conducted over the past few years, including many important aquaculture species, such as catfish (Li et al., 2014), tilapia (Liu et al., 2013), Atlantic salmon (Gonen et al., 2014), as well as common carp. The recently published high density linkage map contains 28,194 SNP markers with an average locus interval of 0.75 cM (Peng et al., 2016). All these resources provide convenience and efficiency for the subsequent studies on evolutionary genomics, ecology, physiology, immunology, as well as the QTLs localization of significant traits.

In the current study, we were interested to unravel the genetic architecture of head size traits in Yellow River carp. For this purpose, we conducted GWAS using 433 Yellow River carp individuals from multiple families to identify loci potentially associated with head size related traits including HL, HBR, ED, and EC. QTL mapping was utilized in a full-sib family of Yellow River carp to improve power and make the result robust against population stratification and admixture. To get a view of candidate genes, we searched the reference genome to identify possible candidate genes surrounding the significant SNPs and QTL regions. Furthermore, KEGG pathway analysis was performed, which could increase our understanding of the potential molecular mechanisms underlying fish head size differentiation. Considering these results can provide some references for the phenotype prediction in common carp populations and can be of great value to future MAS. However, further analyses are needed in order to confirm these results.

## Materials and Methods

### Ethics Statement

This study was approved by the Animal Care and Use committee at College of Ocean and Earth Sciences, Xiamen University. The methods were carried out in accordance with approved guidelines.

### Samples and Phenotypic Data

The Yellow River carp families were constructed at Breeding Station of Henan Academy of Fishery Research, Zhengzhou, China, using parents from different ancestors. The offspring were cultured in 2000 m^2^ pond and fed routinely under standard feeding regime. A total of 474 individuals were randomly collected 18 month post hatch from multiple families.

Traits including BW, BL, HL, EC and ED were measured, HBR was calculated accordingly. A Pearson’s correlation was tested to reflect the relationship between the phenotypic pairs, growth traits were included for comparison with head size traits.

### Genotyping and Quality Control

Genomic DNA was extracted from blood samples using DNeasy 96 Blood and Tissue Kit (Qiagen, Shanghai, China), quantified by Nanodrop-1000 spectrophotometer (Thermo Scientific, Wilmington, DE, United States). After integrity examination with agarose gel electrophoresis, genomic DNA samples were diluted to the final concentration of 50 ng/μl for genotyping hybridization. The genotyping process had been described in previous study (Peng et al., 2016). Briefly, DNA samples were genotyped with common carp 250K SNP array at GeneSeek (Lincoln, NE, United States). Custom perl scripts were used for data cleaning and assessment. Affymetrix Genotyping Console software (version 4.0) was used for quality control and Affymetrix Axiom GT1 algorithm was used for SNP calling. SNPs with call rates greater than 95% were collected for further analysis. All individuals with missing genotypes > 5% and SNPs with missing genotypes > 5%, minor allele frequency < 5% were removed using specific parameters in PLINK, leaving 433 individuals (**Supplementary Table S3**) and 106,959 SNPs for the association analysis. Only the SNPs that are heterozygous in at least one parent and are conformed to Mendelian inheritance were used for QTL mapping. The genetic linkage map contains 28,194 SNP markers with a total length of 14,146 cM and an average marker interval of 0.75 cM (Peng et al., 2016).

### Genome-Wide Association Study

Because the individuals were from several families (**Supplementary Table S3**), sample structure based on a centered relatedness matrix in GEMMA was drawn using SNPs remained after quality control. The first and second dimensions were plotted using R package (**Supplementary Figure S2**).

PLINK was initially used to perform genome-wide association analyses. Genome-wide empirical *p*-values were corrected by 100,000 max(T) permutations. The genomic inflation factor (λ, based on median chisq) ranged from 1.50 to 8.73 for the different phenotypes (**Table 1**), suggesting the population stratification. To adjust for population stratification and relatedness, GWAS was additionally performed using the GEMMA software, in which a centered relatedness matrix estimated from genotypes for each phenotype could be used for association studies. GEMMA implements the Genome-wide Efficient Mixed Model Association algorithm and the GWAS was performed with the relatedness matrix, phenotype and genotype fitted into a univariate linear mixed model as follows:

y=Wα+xβ+u+ε

**Table 1:** Genomic inflation factor (λ) from the initial GWAS in PLINK, PVE or “chip heritability” in GEMMA.

Phenotype	λ	PVE	*SE*(PVE)
Body weight	2.15	0.38	0.10
Body length	1.58	0.39	0.10
HBR	8.73	0.24	0.06
Head length	5.37	0.27	0.07
Eye diameter	5.19	0.29	0.07
Eye cross	1.50	0.13	0.08

*HBR, head length/body length ratio; PVE, phenotypic variance explained.*

where y is a vector of phenotypes, W is a covariate matrix with the vector α of the fixed effects including the intercept, x is a vector of marker genotypes and the effect size of the marker is β, u is a vector of random effects and 𝜀 is a vector of residual errors. Wald frequentist test was chosen to test for significance and Bonferroni threshold for *P* < 0.05 was determined as genome-wide significance. Furthermore, GEMMA estimates the proportion of variance in the phenotypes explained (PVE or “chip heritability”). Manhattan plot representing the −log_10_(*P*-value) and QQ plot expressing the expected and observed p-value were produced using R software.

Given that none of the SNPs from GEMMA reached the Bonferroni threshold for *P* < 0.05, we further tried TASSEL software and performed GWAS with the same batch of data. The Kinship matrix generated from TASSEL was regarded as a covariate to improve statistical power. The mixed liner model (MLM) comprising the Q + K model was used to perform GWAS between phenotypes and genotypes.

In this study, −log_10_(1/n) was used as a threshold because the Bonferroni test (0.05/numbers of SNPs) criterion is extremely strict to be a threshold, considering GWAS is hypothesis generating (Yang et al., 2011; Wang et al., 2012). The threshold of suggestive association was arbitrarily set as −log_10_(*P*) > 4.5.

A within-family test of association might have less power but could control better for possible population stratification (Menzel et al., 2007), therefore we conduct GWAS of G1 family using several models including Multi-locus mixed model GWAS (MLMM) in Mixed Linear Model Analysis in SVS 8.8.1 (Golden Helix)^1^, Genome-wide Efficient Mixed Model in GEMMA and QFAM in PLINK (Purcell et al., 2007) as comparisons to the QTL results.

### QTL Mapping

QTL mapping with head size phenotypes from 103 individuals was performed with the high density genetic linkage map recently published (Peng et al., 2016). QTL mapping was performed using MapQTL 6.0 software with “multiple QTL model (MQM) mapping” method using “Mixture Model” algorithms. LOD significance threshold levels were determined by Permutation Test on the basis of 1,000 permutations for the traits. QTL with LOD scores exceeding the chromosome-wide LOD threshold at *P* < 0.01 or the genome-wide LOD threshold at *P* < 0.05 were considered as significant.

### Candidate Genes

We mapped candidate SNPs and QTL regions onto the common carp genome to identify candidate genes associated with head size traits. Briefly, we screened the ± 50 K bp genome regions surrounding the significant SNPs based on common carp reference genome that released at GeneBank (Accession number: GCF_000951615.1)^2^, and annotated the candidate genes accurately by BLAST against the non-redundant protein database.

For better understand the genetic mechanism, pathway analysis was performed using online tools KEGG^3^ and Omicshare^4^.

## Results and Discussion

### Traits Measurement and Analysis

Phenotypic data used in GWAS were shown in **Supplementary Table S1**. BW of the samples ranged from 815 to 1,895 g. Body length ranged from 30.70 to 46.90 cm. HL was 7.87 ± 0.72 cm (Mean ± SD); HBR was 0.23 ± 0.01; ED was 1.12 ± 0.14 cm; EC was 3.88 ± 0.34 cm. Phenotypic data used in QTL mapping were also listed in **Supplementary Table S1**.

There was a certain degree of phenotypic correlation between phenotypic traits (**Supplementary Figure S1**), and most of them reached a significant level of 0.05, among which, more than half of the correlation coefficients reached a very significant level of 0.01. There was a very strong, positive correlation between BW and body length (*r* = 0.859, *p* < 0.01). HL was moderately correlated with BW (*r* = 0.381, *p* < 0.01) and weakly correlated with body length (*r* = 0.256, *p* < 0.01). There was a weak, negative correlation between HBR and BW (*r* = −0.225, *p* < 0.05), a weak and negative correlation between HBR and body length (*r* = −0.354, *p* < 0.01). A moderate, positive correlation was observed between EC and BW (*r* = 0.515, *p* < 0.01), EC and body length (*r* = 0.518, *p* < 0.01). No strong phenotypic correlations were observed within head size traits, including a moderate correlation between HL and ED (*r* = 0.498, *p* < 0.01), a weak correlation between HL and EC (*r* = 0.312, *p* < 0.01) and a weak correlation between ED and EC (*r* = 0.247, *p* < 0.05). No significant correlation was found between HBR and EC, ED and BW, ED and BW. The results of pairwise comparisons indicated the phenotypic correlations among the related traits, which might suggest the potential relevance underlying the genetic basis, and provide valuable information for the following genetic localization of the related traits.

Head size had been previously studied in catfish, which focused on head length, head width and head depth (Geng et al., 2016). Relevant studied were performed mostly in Songpu mirror carp and the related hybrids, including HL, EC, ED, HBR with few molecular markers (Liu et al., 2009; Wang et al., 2010; Xing et al., 2011; Shan et al., 2014). Related research was rarely reported in the Yellow River carp, except for the recent publication focusing on growth-related traits (Peng et al., 2016). HL was reported to be important for determining the weight of rounded fish (Reis Neto et al., 2012). The direct effect of HL on BW in Large Yellow Croaker was greater than that of body length (Liu et al., 2008). Given that HBR has similarity with HL and could better reflect the meat yield, it is of great interest to investigate the genetic basis of HBR to provide insights into head size determination.

### GWAS and QTL Mapping of Head Size Related Traits

**FIGURE 1:**
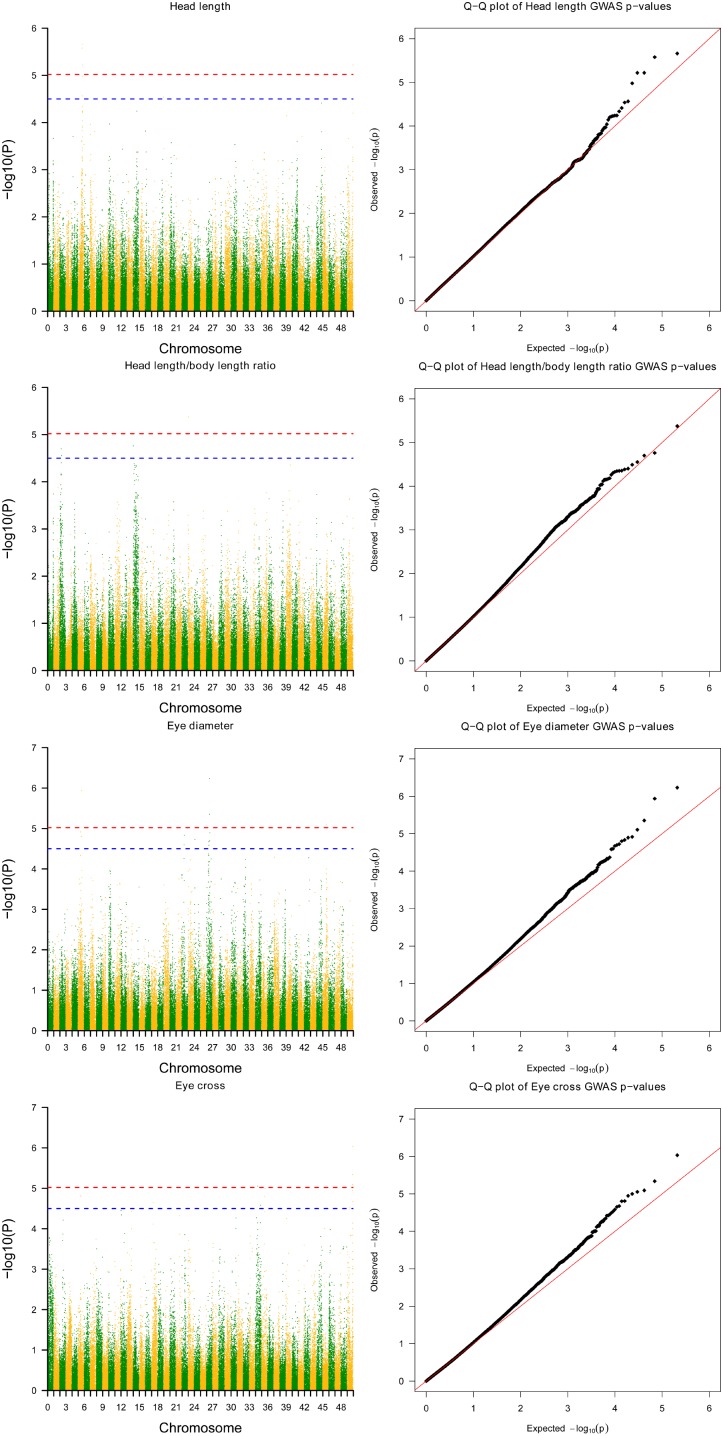
Results from genome-wide association study on head size related traits in Yellow River carp. Significance levels are shown by the red line [–log_10_(1/n) = 5.02] and suggestive the blue line (4.5). HL, head length; HBR, head length/body length ratio; ED, eye diameter; EC, eye cross.

Twelve SNPs reached the genome-wide significance (−log_10_*P*-value > 5.02) in GWAS distributed on 6 linkage groups (LGs), including 4 SNPs for HL, one single for HBR, 4 for ED and 4 for EC (**Table 2** and **Figure 1**). Interestingly, HL and EC shared the same snp166316, with p-value 6.06E-6 and 9.26E-7, respectively. There were 23 SNPs suggestively associated with head size with −log_10(_*P*-value) > 4.5. The phenotypic variance explained (PVE) by genetic factors was no more than 27% for HL, 24% for head/body length ratio, 29% for ED and 13% for EC (**Table 1**).

**Table 2:** *P*-values for significant SNPs from each head size phenotype.

Traits	SNP	LG	Position	*P*-value	Beta *P*	*SE* (Beta *P*)
HL	snp073184	6	21897722	2.65E-06	0.28	0.06
	snp030911	6	21940464	2.19E-06	0.28	0.06
	snp185349	6	23066401	6.05E-06	0.27	0.06
	snp166316	50	18910733	6.06E-06	−0.29	0.06
HBR	snp156398	24	1699825	4.21E-06	0.00	0.00
ED	snp186892	6	19256058	1.16E-06	−0.08	0.02
	snp164489	27	11936777	4.45E-06	0.07	0.02
	snp069279	27	12629625	5.88E-07	0.08	0.02
	snp240725	46	14889833	7.83E-06	0.08	0.02
EC	snp068026	35	10830211	8.83E-06	0.14	0.03
	snp110292	50	17711045	4.58E-06	−0.14	0.03
	snp074034	50	17757647	8.07E-06	−0.14	0.03
	snp166316	50	18910733	9.26E-07	−0.15	0.03

*HL, head length; HBR, head length/ body length ratio; ED, eye diameter; EC, eye cross.*

**FIGURE 2:**
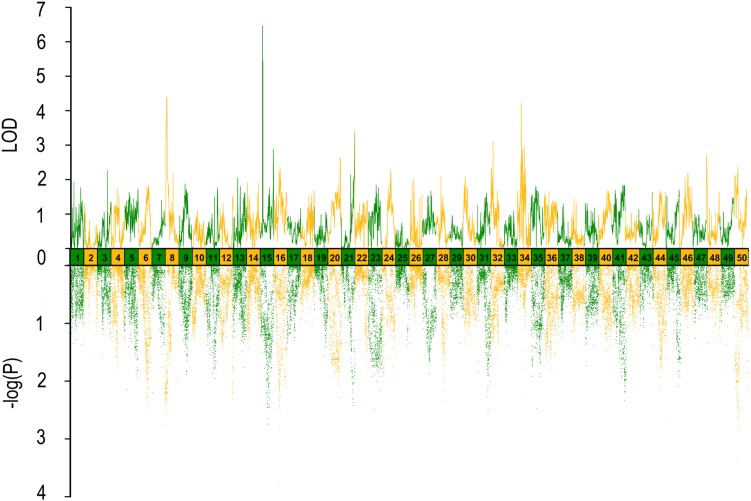
QTL mapping and GWAS of head length in Yellow River carp. Results are based on mapQTL 6 and SVS using a single full-sib family.

**FIGURE 3:**
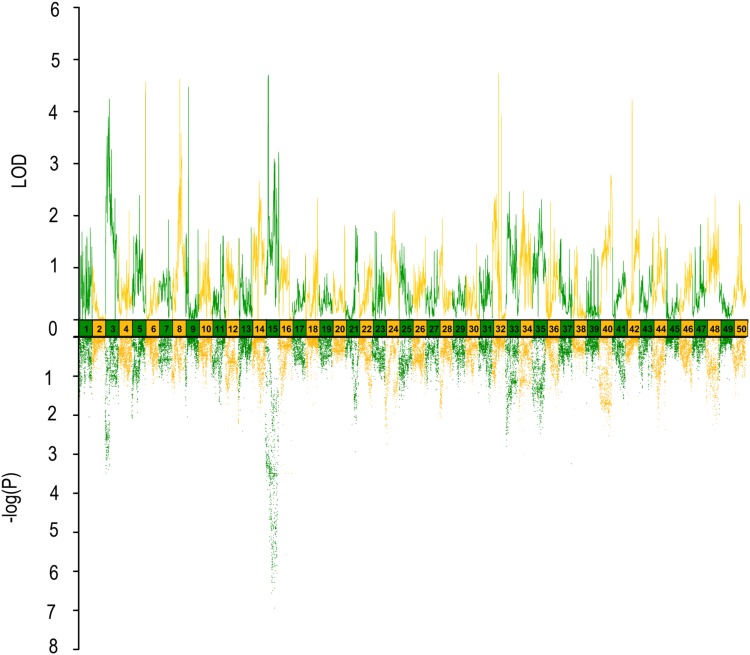
QTL mapping and GWAS of head length/body length ratio in Yellow River carp. Results are based on mapQTL 6 and SVS using a single full-sib family.

**FIGURE 4:**
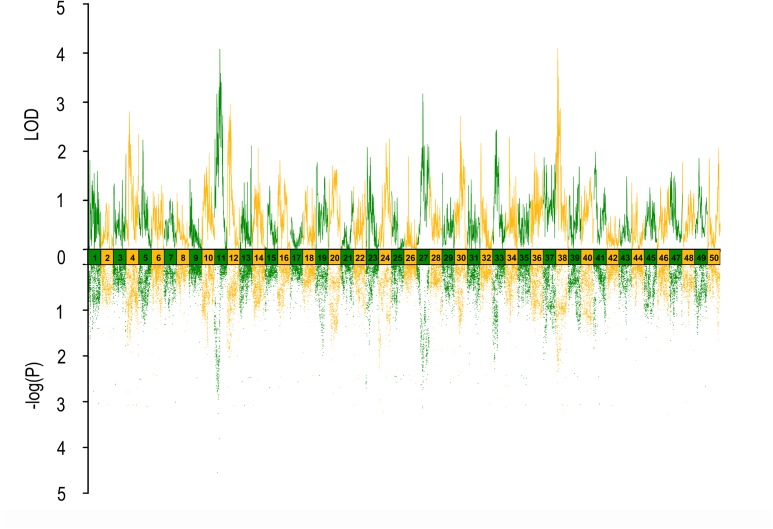
QTL mapping and GWAS of eye diameter in Yellow River carp. Results are based on mapQTL 6 and SVS using a single full-sib family.

**FIGURE 5:**
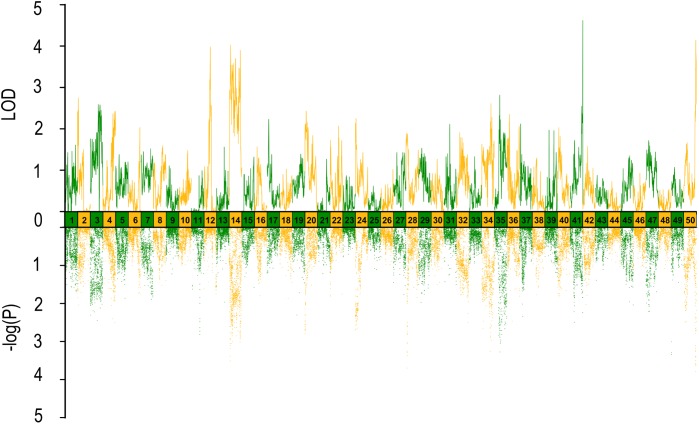
QTL mapping and GWAS of eye cross in Yellow River carp. Results are based on mapQTL 6 and SVS using a single full-sib family.

In QTL mapping, a total of 18 QTL regions associated with head size (**Table 3**) were identified based on chromosome-wide LOD significance with *P* < 0.01. The large distribution of the QTLs suggested the complex genetic control of head size. The 18 QTL regions harbored 151 significant SNPs, including 47 SNPs for HL, 61 SNPs for head/body length ratio, 23 SNPs for ED and 20 for EC. Three QTLs were identified for HL (**Figure 2** and **Table 3**), explaining between 17.4 and 51% of the phenotypic variance. The most significant region was located on LG15 at 29.95–37.31 cM, presenting the highest LOD value of 6.25, explaining the highest total phenotypic variation of 51%. Eight QTLs for HBR were distributed on seven LGs (**Figure 3** and **Table 3**). Surprisingly, HBR15 shared almost the same interval with HL15, demonstrating the similarity between the both traits, which agreed with the result of phenotypic comparison. Another QTL sharing the same LOD score as HBR15 was HBR8, explaining 20.7% of the total PVE. The interval of HBR8, was also the smallest one with a range from 110.97 to 111.27 cM and included 7 SNP markers. Interestingly, the HBR8 shared almost all the SNPs with qBW8a, a QTL responsible for BW detected in our previous study (Peng et al., 2016) (**Supplementary Figure S3**), which strongly proved the correlation between BW and HBR, suggesting that HBR could be an indirect indicator of BW in common carp. While, QTLs for BW shared less with that of HL and HBR. For ED, three QTLs were identified, distributed on two LGs as listed below (**Figure 4** and **Table 3**), explained between 13.4 and 24.2% of the total PVE. Four QTLs were discovered for EC and explained 13.5–21.4% of the total PVE (**Figure 5** and **Table 3**). QTL mapping for the four head size traits showed significant differences among QTL profiles with only a few overlaps, implying different but interactional genetic basis underlying these traits.

**Table 3:** Genomic regions associated with head size in Yellow River carp.

Traits	QTL name	LG	CI(cM)	No. of SNPs SNP	Nearest marker	LOD	Permutation	PVE(%)
HL	HL8	8	13.02–20.25	17	snp061215	4.37	4.2	17.4
	HL15	15	29.95–37.31	29	snp126970	6.25	3.9	51.0
	HL34	34	57.63–57.73	1	snp010428	4.21	4.2	23.7
HBR	HBR3	3	55.53–56.39	7	snp077514	4.24	4.1	15.3
	HBR5	5	237.63–238.43	4	snp173532	4.57	4.4	13.3
	HBR8	8	110.97–111.27	7	snp013043	4.63	4.3	20.7
	HBR9	9	57.64–58.34	1	snp177679	4.41	4.2	51
	HBR15	15	30.91–37.31	28	snp005551	4.63	3.9	45.8
	HBR32	32	123.99–125.19	7	snp158230	4.38	3.9	21
	HBR42a	42	88.69–88.99	2	snp202763	4.03	4.0	20.6
	HBR42b	42	89.63–91.73	5	snp061684	4.22	4.0	20.8
ED	ED11a	11	150.52–152.87	8	snp218634	4.08	3.7	13.4
	ED38	38	34.11–35.88	1	snp078442	4.1	3.6	24.2
	ED38	38	37.27–39.9	14	snp214871	3.84	3.6	19.8
EC	EC12	12	121.07–121.43	1	snp094164	3.63	3.6	13.5
	EC14	14	23.71–25.01	12	snp014385	4.03	3.9	15
	EC41	41	187.64–189.06	1	snp020906	4.63	3.9	21.4
	EC50	50	159.89–166.59	6	snp175214	4.23	3.9	15.2

*HL, head length; HBR, head length/body length ratio; ED, eye diameter; EC, eye cross. PVE, phenotypic variance explained.*

Genome-wide association methodology has recently identified amount of loci for several traits in fish (Geng et al., 2016, 2017). GWAS with dense markers could get closer to the important gene with large enough sample size, but it has a relative high per-sample cost and requires large samples to detect the modest risk effects. QTL mapping in common carp has identified various QTLs associated with head size or other traits (Wang et al., 2010; Shan et al., 2014), some of their results seemed to show some similarity to ours. While, previous studies focusing on head size traits were limited to marker density and could hardly identify traits-related loci accurately. With the popularity of SNP genotyping array, fast, high density and accurate genotyping becomes available in aquaculture specie (Li et al., 2014; Xu J. et al., 2014), which with no doubt facilitate the fine mapping of important traits. The genetic map of common carp provides a dense chromosome framework to correctly order the genome scaffolds and locate QTLs on chromosomes and hence localize more promising candidate genes.

Strategies to increase power of genetic localization include increasing the sample size and marker density or using a comparatively small number of study subjects taken from the extremes of a quantitative distribution (Menzel et al., 2007). Looking back to the GWAS results, none of the markers achieved the Bonferroni threshold of 0.05, which might suggest the less power of our GWAS caused by environment or some other uncertain factors. Then single family based GWAS or QTL analysis could compensate for the deficiency to some extent, making the result robust against population stratification and admixture, both of which can cause potential false-positive associations (Marchini et al., 2004). In fact, the single full-sib family based GWAS using three models all showed high similarity with the result of QTL because of the filtering of population structure and uncertain variables, and MLMM in SVS was remained at last. From this point of view, QTL mapping might be more powerful and allows identification of rare alleles. Combining both the GWAS and QTL would provide more comprehensive understanding of the genetic architecture of head size related traits. It is reasonable that further studies are required to elucidate and confirm the effects of associated loci.

### Candidate Genes Related to Head Size Traits

Fifteen genes were extracted significantly and 32 genes were suggestively associated with head size traits based on GWAS results (**Table 4** and **Supplementary Table S2**). For HL, *parvalbumin* (*parv*) was identified on LG6. Parvalbumin is a muscle protein that aids in relaxation from contraction, patterns of parvalbumin expression determine relaxation rate along the length of the fish (Wilwert et al., 2006). Increased Parvalbumin protein concentration improved both sprint and sustained locomotion in zebrafish (*Danio rerio*), which is linked to fitness and health of animals (Seebacher and Walter, 2012). Furthermore, *parvalbumin* is involved in Insulin-like growth factors (Igf) signaling (Latres et al., 2005). One of the *parvs* was significantly associated with BW and body length in Asian seabass (Xu et al., 2006). Both GWAS and QTL identified the SRSF protein kinases (SRPKs), with *srpk2* on LG50 and *srpk1* on LG15, respectively, suggesting the important effects on HL. *Interleukin-3 receptor class 2 subunit beta* (*il3rb2*) was another gene identified for HL, the elevated expression of which improved hematopoietic recovery and facilitated bone marrow recovery (Chen et al., 2016). Only one gene associated with HBR was identified, named *PH and SEC7 domain-containing protein 1* (*psd*), but the effect on head size remained unclear.

**Table 4:** Candidate genes surround the significant SNPs from GWAS of head size related traits.

Traits	SNP	LG	Gene name	Gene_start	Gene_end	Annotation
Head length	snp073184	6	*parv*	21930290	21932698	Parvalbumin
	snp030911	6	*il3rb2*	21985537	21993706	Interleukin-3 receptor class 2 subunit beta
	snp166316	50	*srpk2*	18957041	18972205	SRSF protein kinase 2
HBR	snp156398	24	*psd*	1637666	1672415	PH and SEC7 domain-containing protein 1
Eye diameter	snp164489	27	*fsrp-5*	11919251	11998304	Follistatin-related protein 5
	snp068026	35	*alpk3*	10821041	10832834	Alpha-protein kinase 3
	snp186892	6	*prrt2*	19299096	19303281	Proline-rich transmembrane protein 2
	snp240725	46	*slco4a1*	14889976	14901592	Solute carrier organic anion transporter family member 4A1
Eye cross	snp068026	35	*frmd5*	10849629	10943834	FERM domain-containing protein 5
	snp110292	50	*gramd2*	17738573	17757106	GRAM domain-containing protein 2
	snp166316	50	*srpk2*	18957041	18972205	SRSF protein kinase 2

*HBR, head length/body length ratio.*

For ED, *follistatin-related protein 5* was identified on LG27. *Follistatin-related genes* are major negative regulators of myostatin *in vivo* and could block some bone morphogenetic proteins (Amthor et al., 2002; Hill et al., 2002). Overexpression of *follistatin* in skeletal muscle of mice could result in double-muscle phenotype (Lee and Mcpherron, 2001). Suggestively associated genes including *fibroblast growth factor receptor-like 1* (*fgfrl1*), which is a member of the fibroblast growth factor receptor family that controls the formation of musculoskeletal tissues and skull (Iseki et al., 1999; Beyeler and Trueb, 2006). *Srpk2* was identified both for HL and EC in GWAS, revealing the overlapping genetic control of the both traits.

**FIGURE 6:**
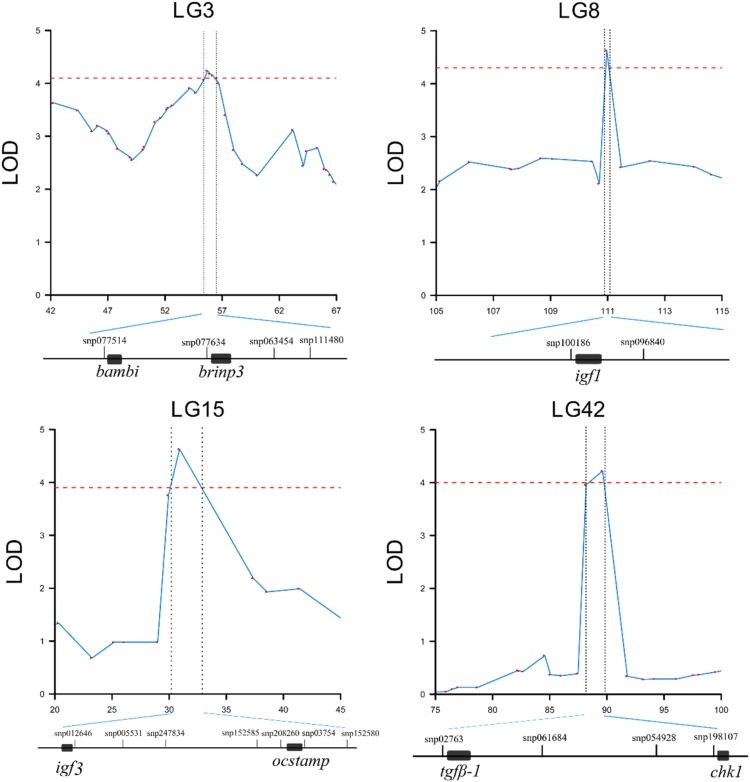
QTL region and candidate genes for head length/body length ratio in Yellow River carp. Genes are extracted in the ± 50 K bp genome regions around the significant SNPs. Black rectangles represent the candidate genes.

A total of 193, 223, 56, and 73 genes were identified for HL, HBR, ED and EC, respectively in QTL mapping (**Table 5** and **Figure 6**). Pathway analysis showed that 176 of these genes were enriched in several related pathways, including FOXO signaling pathway, TGF-beta signaling pathway, WNT signaling pathway, and so on (**Supplementary Figures S4**–**S10**). HL15 and HBR15 shared almost all the candidates. Interestingly, we identified *igf3* in HL15 and HBR15, explaining relatively high total phenotypic variations. Significant attention has been paid to the functions of the fish specific gene, *igf3* in gonad development and its interaction with growth hormone pathways (Wang et al., 2008; Nelson and Van Der Kraak, 2010; Wan and Chan, 2010; Li et al., 2015). The candidate gene *nras* identified in HL15 belongs to small GTPases, which were important for head formation in early Xenopus development (Wunnenberg-Stapleton et al., 1999). We also identified *igf1* in the region of HBR8, which was previously reported to be possibly associated with BW in common carp (Peng et al., 2016), revealing the genetic overlap between growth related traits and head size traits. *Igf1* is also an excellent candidate gene closely associated with body size, birth weight, head formation and head size (Richard-Parpaillon et al., 2002; Sutter et al., 2007; Reynaud et al., 2010; Forman, 2013; Geng et al., 2016). Thereafter, we could speculate that Igf signaling pathway might show more potential for body growth and head size in common carp by cross talk with other related signaling, for example FOXO signaling pathway (**Supplementary Figure S5**).

**Table 5:** Summary of candidate genes from QTLs for head size in Yellow River carp.

Traits	QTL	LG	Gene name	Gene start	Gene end	Annotation
HL	HL8	8	*mGluR8*	2862631	2924019	Metabotropic glutamate receptor 8
	HL15	15	*igf3*	6453808	6456799	Insulin-like growth factor 3
			*nras*	3772910	3775277	GTPase NRas
	HL34	34	*slc8a1*	9307807	9389516	Sodium/calcium exchanger 1
HBR	HBR3	3	*bambi*	6831883	6835733	BMP and activin membrane-bound inhibitor homolog
			*brinp3*	15599984	15649304	BMP/retinoic acid-inducible neural-specific protein 3
	HBR5	5	*cd62l*	26777327	26799978	L-selectin
			*tcf20*	26859180	26869590	Transcription factor 20
	HBR8	8	*igf1*	10938104	10948207	Insulin-like growth factor I
			*apaf1*	11240066	11267749	Apoptotic protease-activating factor 1
	HBR9	9	*astn2*	3666252	3758201	Astrotactin-2
	HBR15	15	*igf3*	6453808	6456799	Insulin-like growth factor 3
			*pdzd4*	11133352	11148182	PDZ domain-containing protein 4
			*notch2*	9664850	9698111	Neurogenic locus notch homolog protein 2
			*ocstamp*	12867795	12872198	Osteoclast stimulatory transmembrane protein
	HBR32	32	*grb10*	14901703	14966162	Growth factor receptor-bound protein 10
	HBR42a	42	*tgfb1*	9282798	9286738	Transforming growth factor beta-1
	HBR42b		*chk1*	9822817	9825726	Serine/threonine-protein kinase Chk1
			*il7r-α*	9767788	9769952	Interleukin-7 receptor subunit alpha
			*skp2*	9738127	9743090	S-phase kinase-associated protein 2
ED	ED11	11	*espl1*	8449618	8461291	Separin
EC	EC12	12	*pik3r3*	18123344	18135519	Phosphatidylinositol 3-kinase regulatory subunit gamma
			*pik3r1*	17996051	18000702	Phosphatidylinositol 3-kinase regulatory subunit alpha
	EC14	14	*igf1r*	5032149	5099651	Insulin-like growth factor 1 receptor
			*irs1*	7026279	7029442	Insulin receptor substrate 1-B
	EC50	50	*sfrp2*	17938832	17941016	Secreted frizzled-related protein 2

*HL, head length; HBR, head length/body length ratio; ED, eye diameter; EC, eye cross.*

BMP an*d activin membrane-bound inhibitor homolog* (*bambi*) was identified as a candidate gene in the genome region of HBR3. Bambi is a BMP signaling transmembrane glycoprotein and functions as a negative regulator of TGF-β signaling during development (Sekiya et al., 2004) (**Supplementary Figure S6**). Thus, *bambi* is seemingly to have role in shaping the HBR in Yellow River carp through bone or growth regulation. On LG15, we also identified *neurogenic locus notch homolog protein 2* (*notch2*) in the QTL region of HBR15, which was in the Grk/Egfr pathway (**Supplementary Figure S8**) and played critical roles in regulation of bone mass (Simpson et al., 2011), hence may leading to some effects on HBR. As for HBR32, *growth factor receptor-bound protein 10* (*grb10*, also known as *insulin receptor-binding protein*) was identified, which was an interacting partner of the *igf1 receptor* (*igf1r*) and *insulin receptor* (*ir*) (Dey et al., 1996; Vecchione et al., 2003). It could be speculated that the binding of *grb10* to *igf1r* inhibits the binding of *igf1* to the ligand *igf1r* and then affects the Igf signaling, leading an impact on head size.

For ED, we identified *separin* (*esp1*), a gene proposed to be required for embryonic anterior-posterior axis formation together in *Caenorhabditis elegans* (Rappleye et al., 2002). In ED38, *glycogen synthase kinase-3 beta* (*gsk3b*) was identified (**Supplementary Figure S9**), which functions as a negative regulator of IL-17-mediated inflammatory responses, and was depended by IL-17-induced chemokine expression enhanced by *igf1*, thus involved in Obesity (Ge et al., 2013). For EC, we identified *igf1r* in EC14. A non-synonymous mutation in *igf1r* contributed to reduce size in dogs (Hoopes et al., 2012). In EC50, *secreted frizzled-related protein 2* (*sfrp2*) was annotated. *Sfrp2* was a Wnt signaling antagonist with known function during eye lens development (Lin et al., 2007; Sugiyama et al., 2013) and potential functions on body conformation in catfish (Geng et al., 2017). Certainly, some of these genes within QTL regions might have unknown but related functions.

Overall, both the literature data and our present study show that Igf signaling pathways and some other signaling pathways related to bone and muscle development may play crucial roles in head formation and head size determination together with body growth. Genetic modification and selection of the related loci or genes may subsequently affect the phenotypes and provide more insights in the future molecular marker assisted breeding.

## Conclusion

Here we performed GWAS and QTL mapping strategies to identify loci and genes associated with head size. GWAS identified 12 SNPs significantly associated with head size. A total of 18 QTLs were identified in QTL mapping, revealing the polygenic nature of these complex traits. Candidate genes were identified around the significant SNPs, including *parv*, *fsrp5*, *igf1* and *igf3*, *grb10*, *igf1r*, *sfrp2*, indicating the importance of Igf signaling as well as some other bone and muscle development related signaling pathways in head size determination. The combination of both GWAS and QTL provides more understanding for fish head formation and could be considered in genetic breeding program on common carp for economical purpose on both carcass yield and ornamental value.

## Data Availability

The common carp reference genome used in this study is accessible either at CarpBase (http://www.carpbase.org) in the Help/Download section or in the GeneBank with Accession number of GCF_000951615.1 (https://www.ncbi.nlm.nih.gov/assembly/GCF_000951615.1/). All raw sequencing data have been deposited in the NCBI Sequence Read Archive (SRA: SRP026407).

## Author Contributions

PX conceived and supervised the study. LC analyzed the data and drafted the manuscript. WP and BC helped on data analysis. SK, FP, and ZZ helped on manuscript preparation. JF helped on carp family construction and sample collection. PX and XL revised the manuscript. All the authors have read and approved the manuscript.

## Conflict of Interest Statement

The authors declare that the research was conducted in the absence of any commercial or financial relationships that could be construed as a potential conflict of interest.

## Footnotes

1 http://www.goldenhelix.com

2 https://www.ncbi.nlm.nih.gov/assembly/GCF_000951615.1/

3 http://www.genome.jp/kegg/pathway.html

4 http://www.omicshare.com/tools/Home/Soft/pathwaygsea

## Supplementary Material

FIGURE S1 Phenotypic correlation of the related traits in Yellow River carp. Circle size was distinguished by correlation coefficient; x indicate no correlation.

FIGURE S2 Sample structure for GWAS identified by a centered relatedness matrix in GEMMA. The first dimension (mds 1) was assigned to *X* axis, and the second dimension (mds 2) was assigned to *Y* axis. Individuals from G1 (the QTL family) were circled in pink.

FIGURE S3 Comparison of growth traits and head size traits in Yellow River carp. HL, head length; HBR, head length/body length ratio; BW, body weight; BL, body length.

FIGURE S4 KEGG pathway enrichment of the candidates from QTL. Gene number was assigned to *X* axis, pathways were assigned to *Y* axis.

FIGURE S5 QTL candidates in FOXO signaling pathway. The candidates were marked in red. The plots were drawn using KEGG online tools.

FIGURE S6 QTL candidates in TGF-BETA signaling pathway. The candidates were marked in red. The plots were drawn using KEGG online tools.

FIGURE S7 QTL candidates in WNT signaling pathway. The candidates were marked in red. The plots were drawn using KEGG online tools.

FIGURE S8 QTL candidates in Grk/Egfr pathway. The candidates were marked in red. The plots were drawn using KEGG online tools.

FIGURE S9 QTL candidates in INSULIN signaling pathway. The candidates were marked in red. The plots were drawn using KEGG online tools.

FIGURE S10 QTL candidates in CALCIUM signaling pathway. The candidates were marked in red. The plots were drawn using KEGG online tools.

TABLE S1 Phenotypic data of Yellow River carp in this study. Min, minimum; Max, maximum; SD, standard deviation.

TABLE S2 Genes suggestively associated with head size of Yellow River carp. HL, head length; HBR, head length/body length ratio; ED, eye diameter; EC, eye cross.

TABLE S3 Individuals and family information of common carp. NA: information lost.
