# Supplementary figures and images for: Genetic Mapping of Head Size Related Traits in Common Carp (Cyprinus carpio)
Source: Front Genet. 2018 Oct 9;9:448. doi: 10.3389/fgene.2018.00448 (PMC6190898; doi:10.3389/fgene.2018.00448)

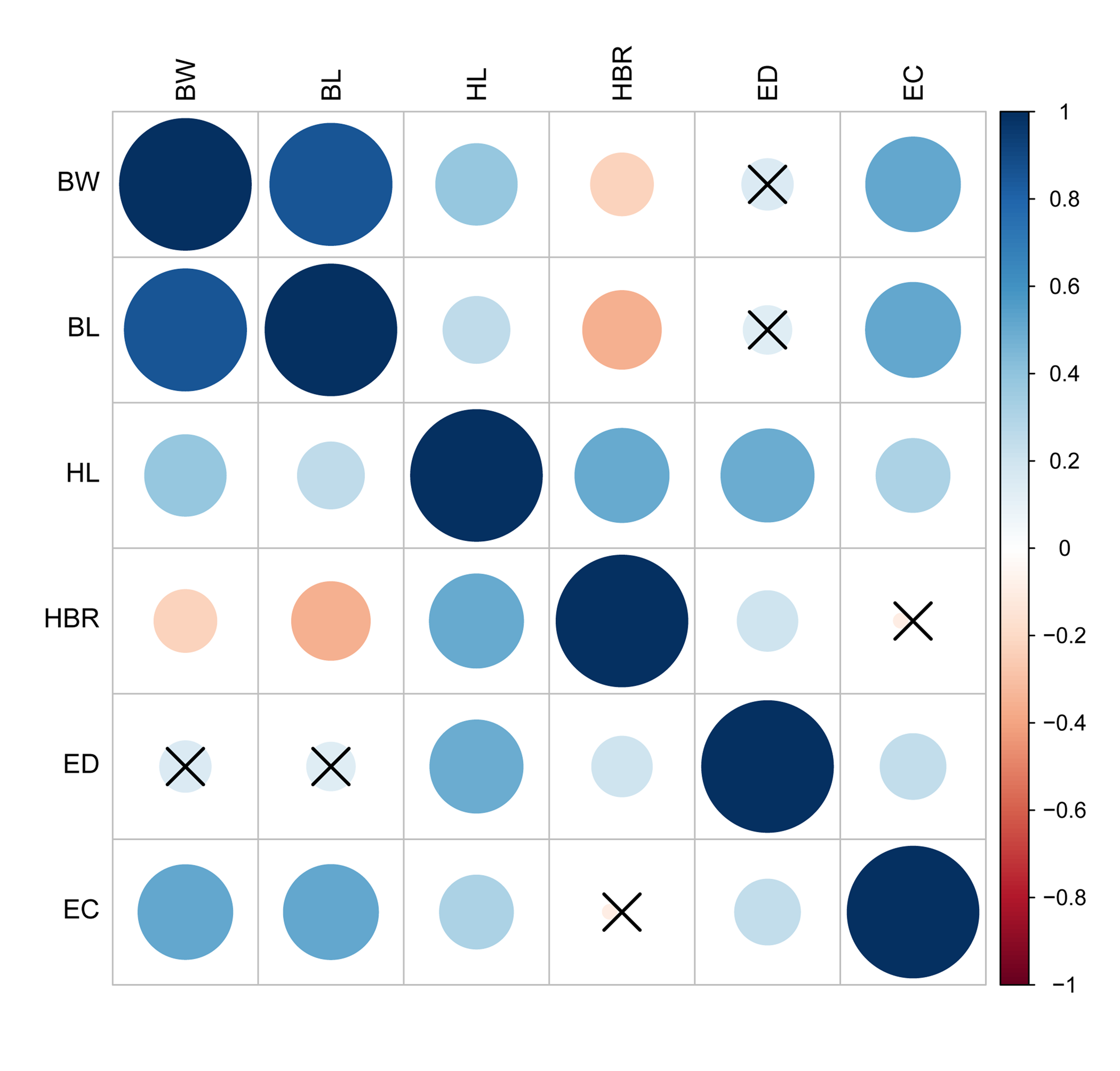

Supplement: FIGURE S1 — Phenotypic correlation of the related traits in Yellow River carp. Circle size was distinguished by correlation coefficient; x indicate no correlation. [file Image_1.TIF]

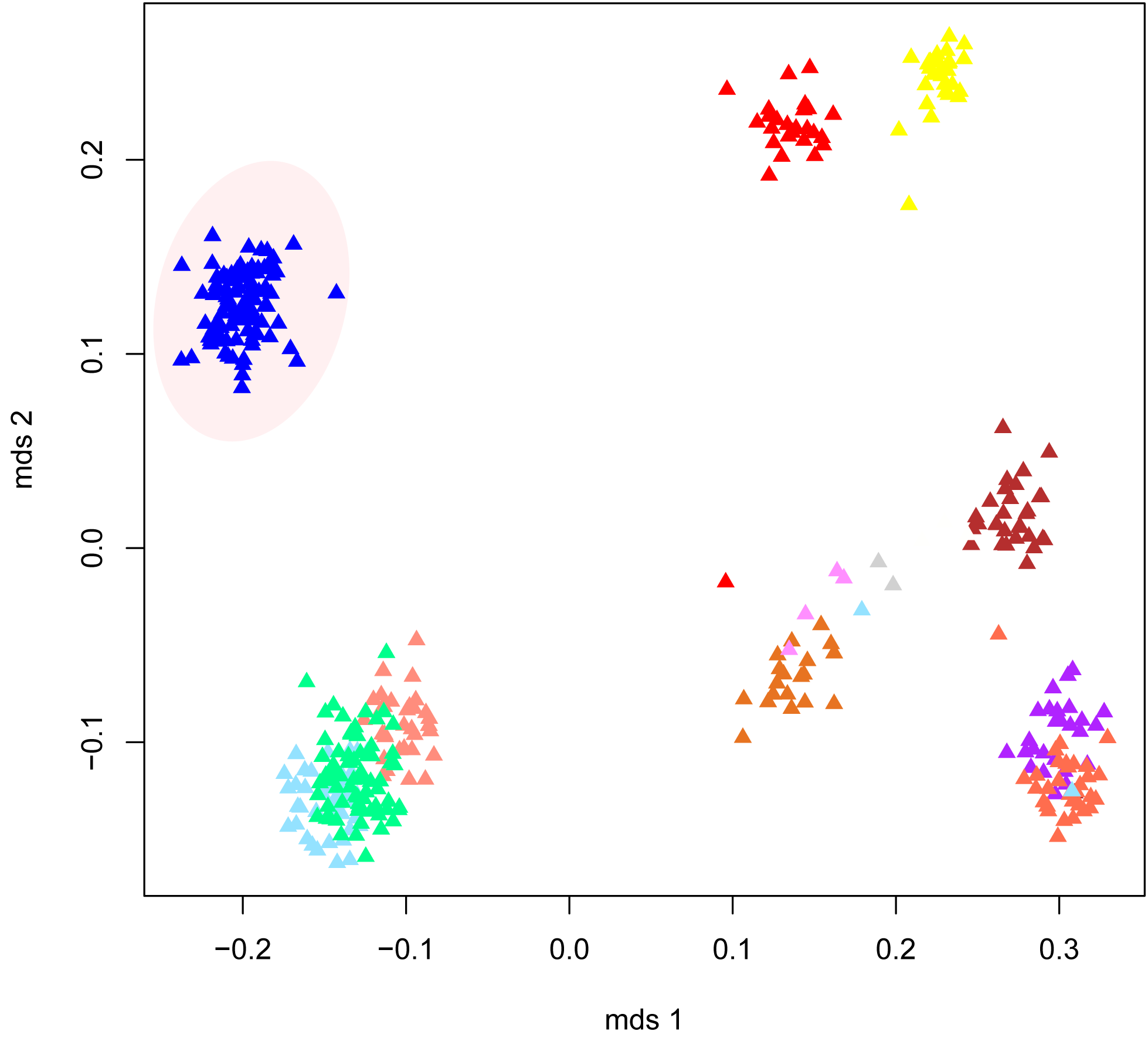

Supplement: FIGURE S2 — Sample structure for GWAS identified by a centered relatedness matrix in GEMMA. The first dimension (mds 1) was assigned to X axis, and the second dimension (mds 2) was assigned to Y axis. Individuals from G1 (the QTL family) were circled in pink. [file Image_2.TIF]

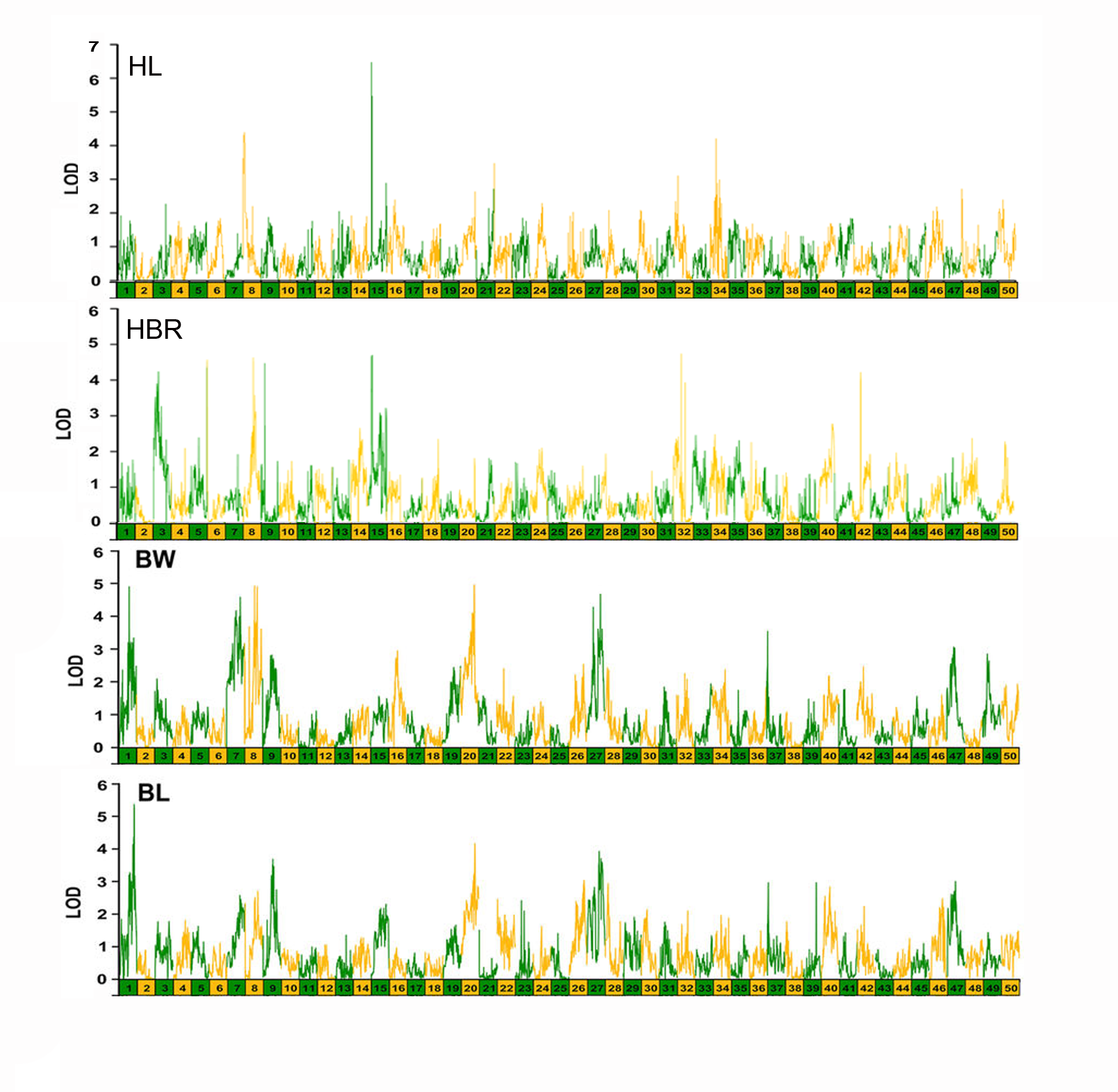

Supplement: FIGURE S3 — Comparison of growth traits and head size traits in Yellow River carp. HL, head length; HBR, head length/body length ratio; BW, body weight; BL, body length. [file Image_3.TIF]

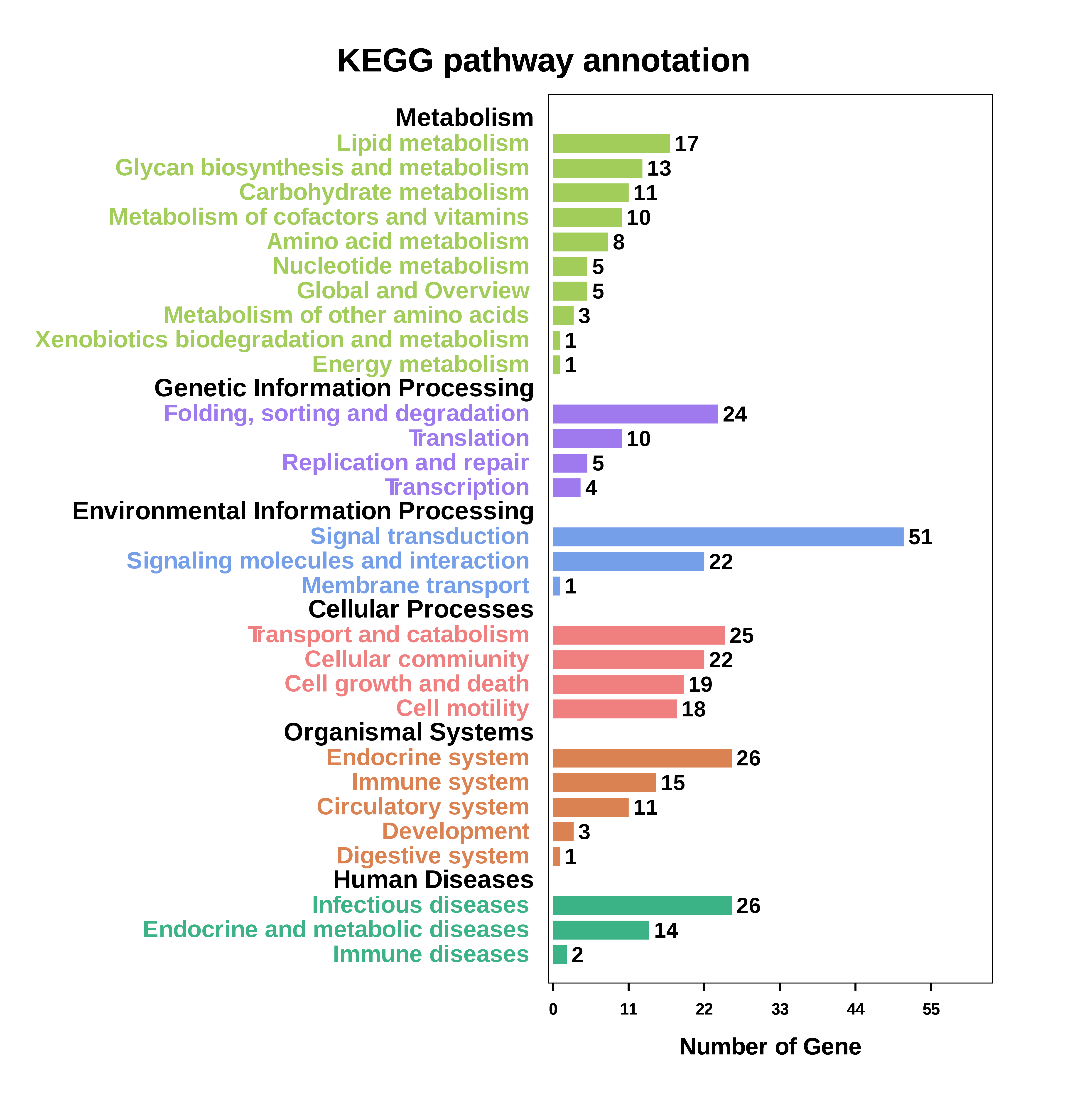

Supplement: FIGURE S4 — KEGG pathway enrichment of the candidates from QTL. Gene number was assigned to X axis, pathways were assigned to Y axis. [file Image_4.TIF]

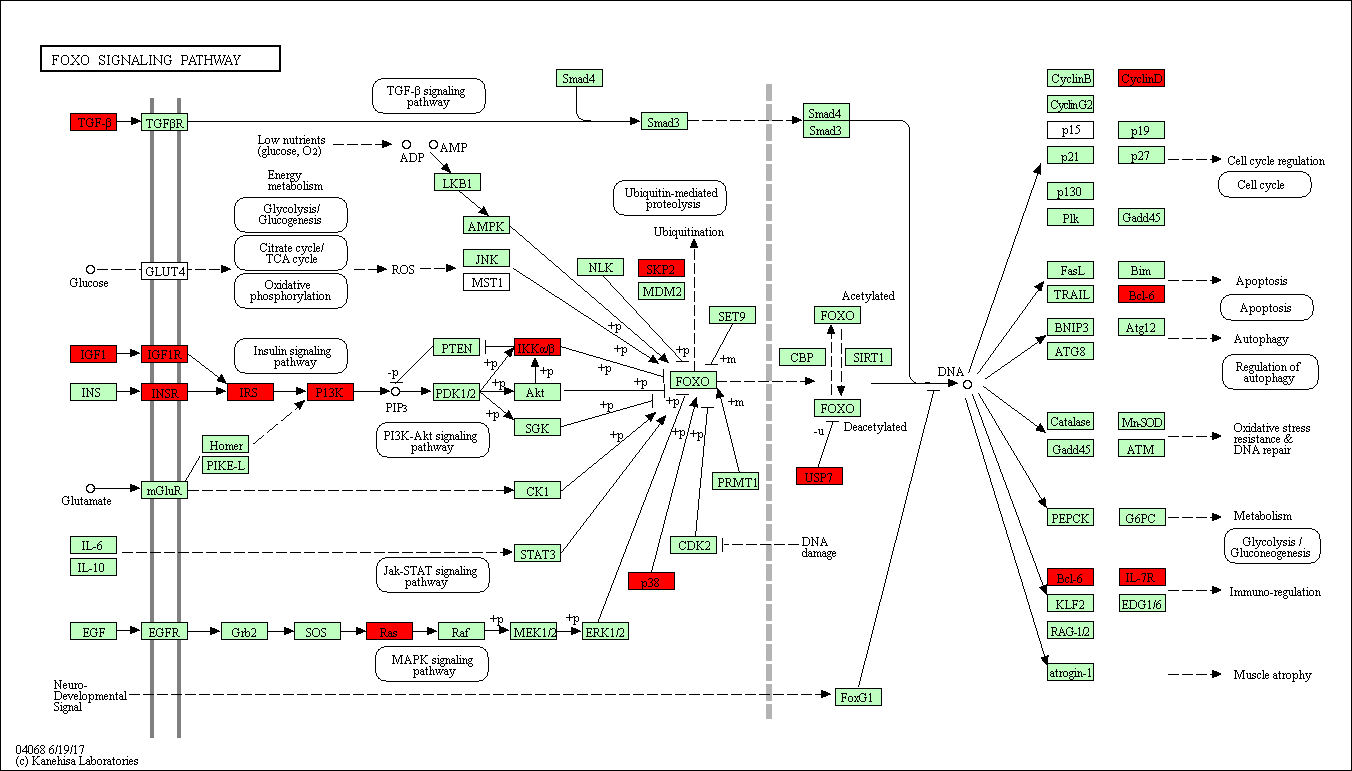

Supplement: FIGURE S5 — QTL candidates in FOXO signaling pathway. The candidates were marked in red. The plots were drawn using KEGG online tools. [file Image_5.PNG]

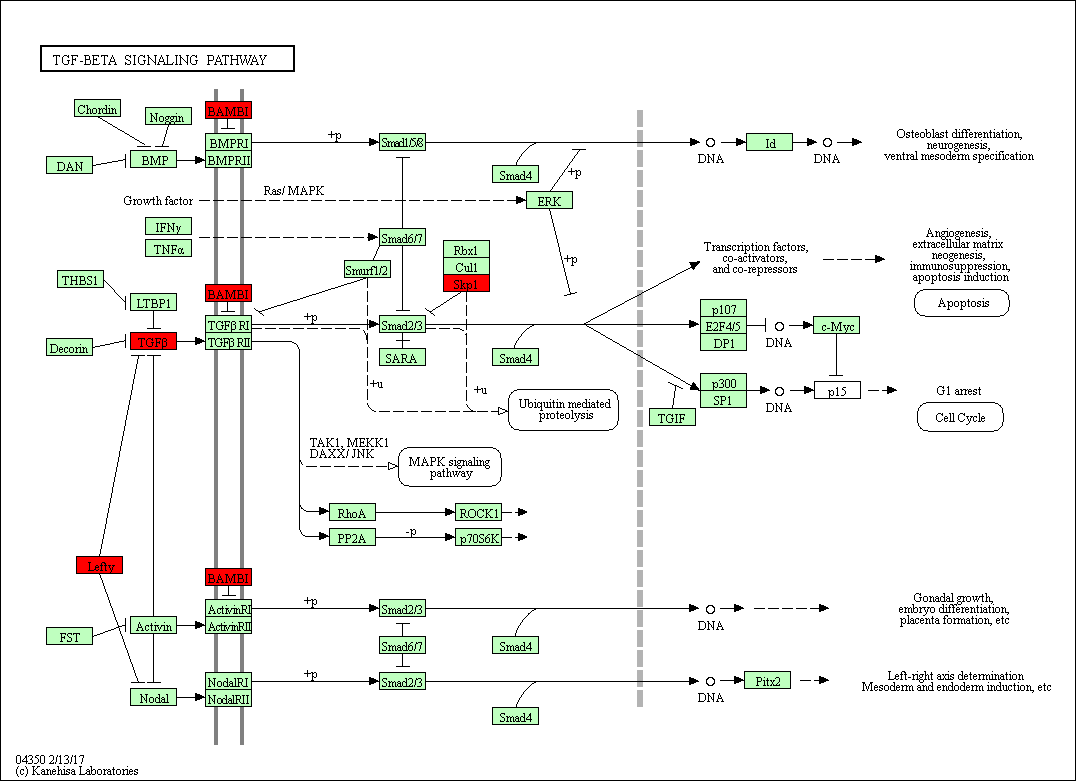

Supplement: FIGURE S6 — QTL candidates in TGF-BETA signaling pathway. The candidates were marked in red. The plots were drawn using KEGG online tools. [file Image_6.PNG]

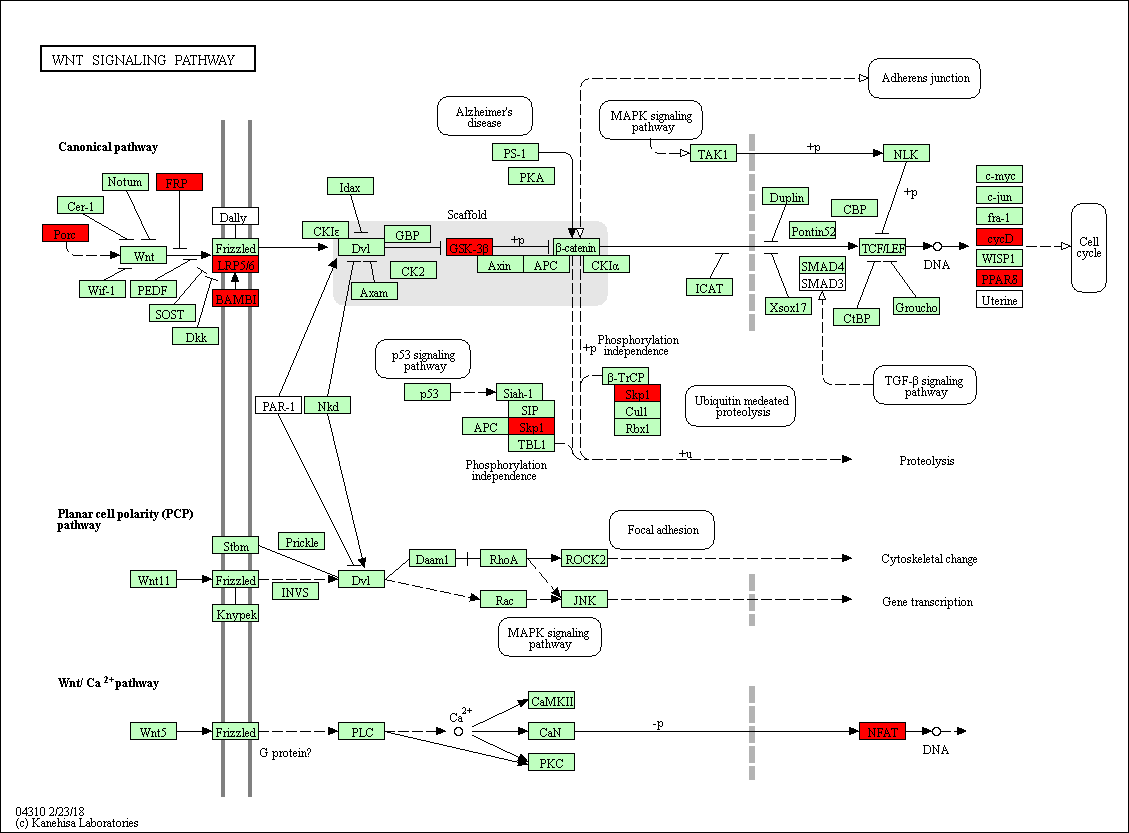

Supplement: FIGURE S7 — QTL candidates in WNT signaling pathway. The candidates were marked in red. The plots were drawn using KEGG online tools. [file Image_7.PNG]

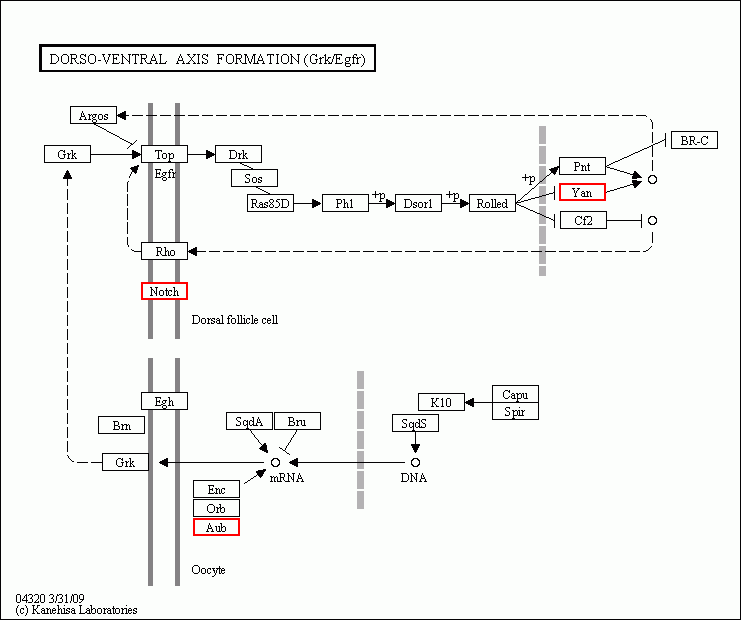

Supplement: FIGURE S8 — QTL candidates in Grk/Egfr pathway. The candidates were marked in red. The plots were drawn using KEGG online tools. [file Image_8.PNG]

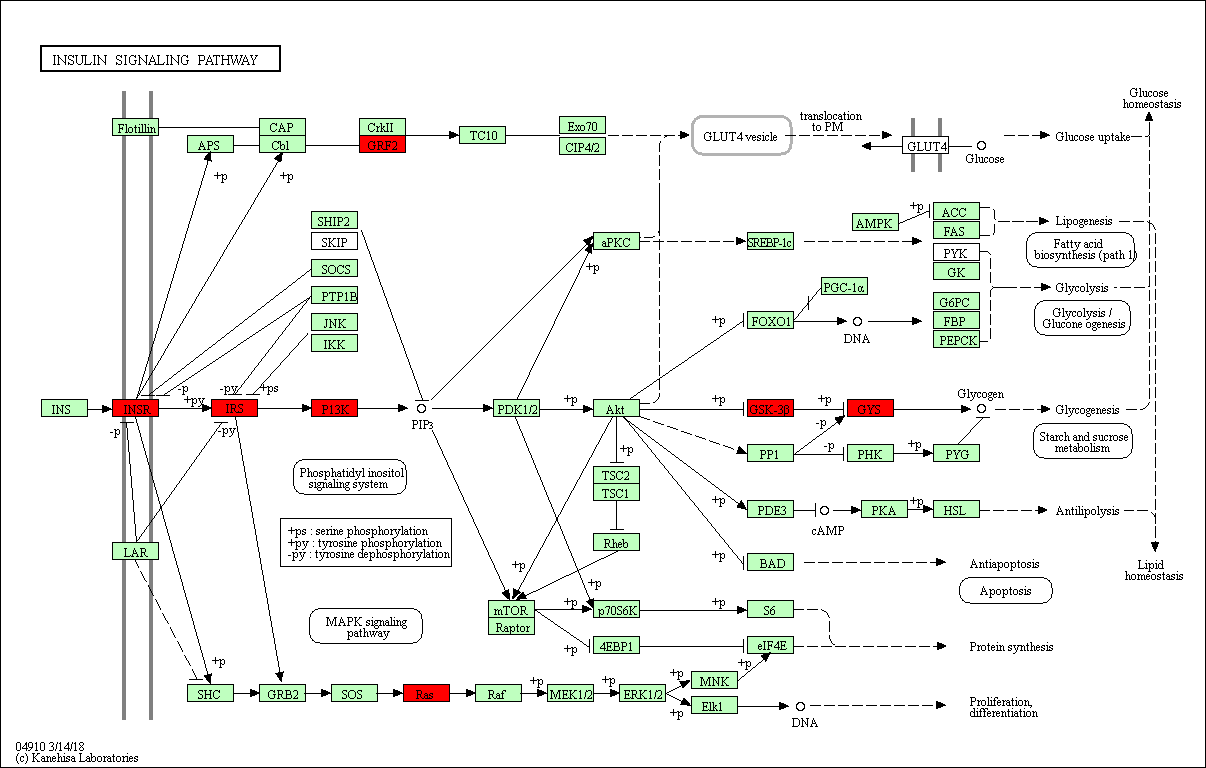

Supplement: FIGURE S9 — QTL candidates in INSULIN signaling pathway. The candidates were marked in red. The plots were drawn using KEGG online tools. [file Image_9.PNG]

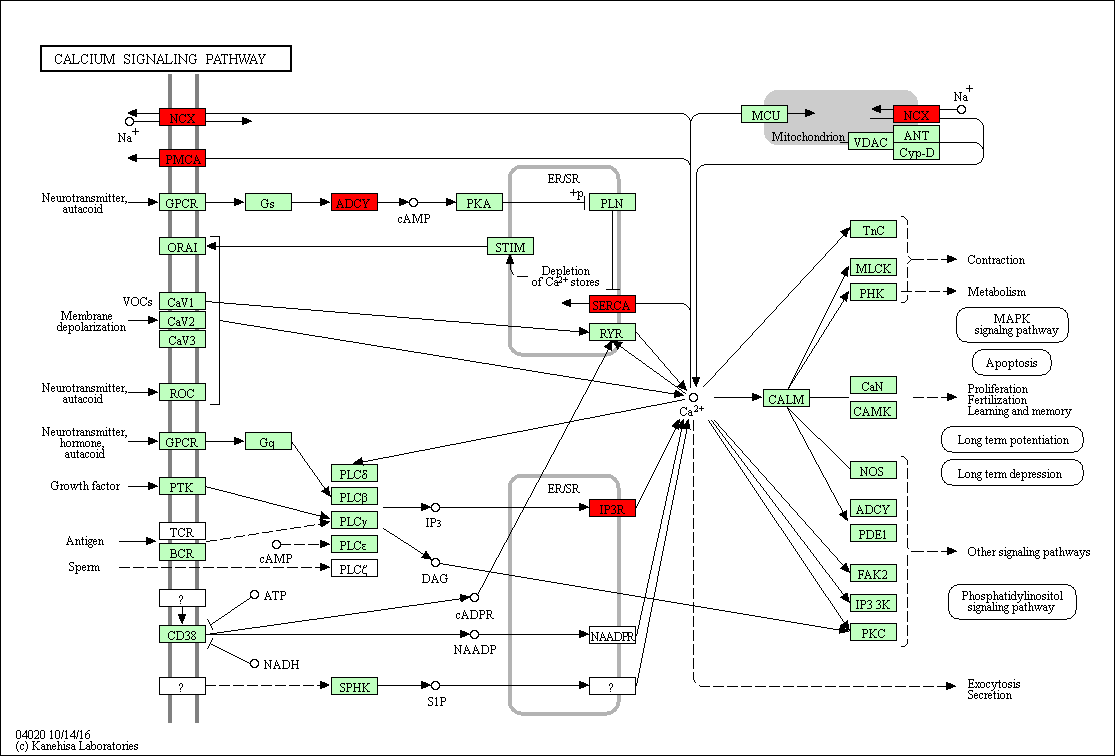

Supplement: FIGURE S10 — QTL candidates in CALCIUM signaling pathway. The candidates were marked in red. The plots were drawn using KEGG online tools. [file Image_10.PNG]
