# Supplementary material for: Genetic Mapping of Head Size Related Traits in Common Carp (Cyprinus carpio)
Source: Front Genet. 2018 Oct 9;9:448. doi: 10.3389/fgene.2018.00448 (PMC6190898; doi:10.3389/fgene.2018.00448)
Supplement: TABLE S1 — Phenotypic data of Yellow River carp in this study. Min, minimum; Max, maximum; SD, standard deviation. [file Table_1.docx]

**Genetic mapping of head size related traits in common carp (*Cyprinus carpio*)**

Lin Chen^1,2^, Wenzhu Peng^1^, Shengnan Kong^1,2^, Fei Pu^1^, Baohua Chen^1^, Zhixiong Zhou^1^, Jianxin Feng^3^, Xuejun Li^2^, Peng Xu^1,4,5*^

*Correspondence:

Peng Xu

e-mail: xupeng77@xmu.edu.cn. Tel.:86-592-2880812(O)

SUPPLEMENTARY TABLES

**S1 Table Phenotypic data of Yellow River carp in this study.** Min, minimum; Max, maximum; SD, standard deviation.

| Programs | Traits | N | Min | Max | Mean | SD |
| --- | --- | --- | --- | --- | --- | --- |
| GWAS | BW (g) | 433 | 815 | 1895 | 1356.28 | 211.96 |
|  | BL (cm) | 433 | 30.70 | 46.90 | 37.81 | 2.21 |
|  | HL (cm) | 433 | 6.14 | 10.31 | 7.87 | 0.72 |
|  | HBR | 433 | 0.19 | 0.27 | 0.23 | 0.01 |
|  | ED (cm) | 433 | 0.77 | 1.64 | 1.12 | 0.14 |
|  | EC (cm) | 433 | 2.53 | 4.82 | 3.88 | 0.34 |
| QTL | BW (g) | 103 | 820 | 1840 | 1361.84 | 184.16 |
|  | BL (cm) | 103 | 32.53 | 46.54 | 38.02 | 2.38 |
|  | HL (cm) | 103 | 6.14 | 10.31 | 8.31 | 0.76 |
|  | HBR | 103 | 0.21 | 0.27 | 0.24 | 0.01 |
|  | ED (cm) | 103 | 0.87 | 1.71 | 1.20 | 0.14 |
|  | EC (cm) | 103 | 3.14 | 5.24 | 3.96 | 0.36 |
