# Supplementary material for: Genetic Mapping of Head Size Related Traits in Common Carp (Cyprinus carpio)
Source: Front Genet. 2018 Oct 9;9:448. doi: 10.3389/fgene.2018.00448 (PMC6190898; doi:10.3389/fgene.2018.00448)
Supplement: TABLE S2 — Genes suggestively associated with head size of Yellow River carp. HL, head length; HBR, head length/body length ratio; ED, eye diameter; EC, eye cross. [file Table_2.docx]

**Genetic mapping of head size related traits in common carp (*Cyprinus carpio*)**

Lin Chen^1,2^, Wenzhu Peng^1^, Shengnan Kong^1,2^, Fei Pu^1^, Baohua Chen^1^, Zhixiong Zhou^1^, Jianxin Feng^3^, Xuejun Li^2^, Peng Xu^1,4,5*^

*Correspondence:

Peng Xu

e-mail: xupeng77@xmu.edu.cn. Tel.:86-592-2880812(O)

SUPPLEMENTARY TABLES

**S2 Table Genes suggestively associated with head size of Yellow River carp.** HL, head length; HBR, head length/body length ratio; ED, eye diameter; EC, eye cross.

| Traits | SNP | LG | P-value | SNP position | Gene start | Gene end | Annotation |
| --- | --- | --- | --- | --- | --- | --- | --- |
| HL | snp217970 | 6 | 2.72E-05 | 24200673 | 24223708 | 24250245 | TOM1-like protein 2 |
| HL | snp048954 | 20 | 2.90E-05 | 566635 | 611598 | 613704 | Histidine triad nucleotide-binding protein 1 |
| HBR | snp111484 | 3 | 2.00E-05 | 6949890 | 6989110 | 7048267 | CMP-N-acetylneuraminate-beta-1,4-galactoside alpha-2,3-sialyltransferase |
| HBR | snp078753 | 3 | 2.80E-05 | 8893557 | 8878674 | 8990546 | Disks large homolog 1 |
| HBR | snp005194 | 15 | 1.73E-05 | 1349140 | 1382539 | 1467840 | Homeobox protein cut-like 2 |
| ED | snp055556 | 6 | 1.22E-05 | 14395100 | 14412287 | 14444288 | Retinoic acid receptor alpha-B |
| ED | snp156189 | 6 | 1.58E-05 | 18292955 | 18335850 | 18353946 | ATP-citrate synthase |
| ED | snp083385 | 23 | 2.53E-05 | 8260481 | 8293846 | 8340535 | Actin-binding LIM protein 1 |
| ED | snp025046 | 23 | 1.47E-05 | 9132752 | 9178297 | 9179379 | Urotensin-2 receptor |
| ED | snp198347 | 25 | 1.90E-05 | 3343962 | 3339849 | 3404800 | Collagen alpha-1(XXI) chain |
| ED | snp247183 | 27 | 2.12E-05 | 9841752 | 9875595 | 9885753 | Cleavage stimulation factor subunit 2 |
| ED | snp247223 | 27 | 2.60E-05 | 9954918 | 9977729 | 10006983 | Fibroblast growth factor receptor-like 1 |
| ED | snp171433 | 27 | 2.01E-05 | 13233953 | 13282788 | 13317539 | PDZ and LIM domain protein 7 |
| ED | snp248049 | 27 | 1.28E-05 | 13680466 | 13711347 | 13737612 | Diacylglycerol kinase theta |
| EC | snp179418 | 6 | 1.55E-05 | 14562832 | 14608784 | 14612743 | Primary amine oxidase |
| EC | snp140906 | 18 | 2.23E-05 | 8391717 | 8384595 | 8455108 | Acid-sensing ion channel 4 |
| EC | snp046305 | 35 | 2.62E-05 | 23572888 | 23615157 | 23619364 | MOB kinase activator 2 |
| EC | snp149968 | 36 | 1.57E-05 | 12755838 | 12794911 | 12810007 | Proline-serine-threonine phosphatase-interacting protein 1 |
| EC | snp207656 | 36 | 9.99E-06 | 15224035 | 15176010 | 15207424 | Genetic suppressor element 1 |
| EC | snp052001 | 50 | 2.96E-05 | 8844794 | 8878955 | 8901715 | Inositol hexakisphosphate and diphosphoinositol-pentakisphosphate kinase 2 |
| EC | snp011319 | 50 | 1.13E-05 | 17859441 | 17882353 | 17914014 | Protogenin B (Fragment) |
| EC | snp203717 | 50 | 2.12E-05 | 18706371 | 18667942 | 18710927 | Neuronal cell adhesion molecule |
